# Effect of Ramadan fasting on thyroid functions in hypothyroid patients taking levothyroxine: a systematic review and meta-analysis

**DOI:** 10.1007/s11845-023-03526-z

**Published:** 2023-09-21

**Authors:** Mohamed Mohamed Belal, Asmaa Reda Youssef, Hany Baker, Nessreen Awaad Elalaky, Ahmed Atef Marey, Muhammed Adel Quaisy, Eslam Mohammed Rabea

**Affiliations:** Faculty of Medicine, Alexandria University, Alexandria, Egypt

**Keywords:** Fasting, Hypothyroidism, Levothyroxine, Ramadan, TSH

## Abstract

**Background:**

The major changes in the timing of meals during Ramadan may be challenging for hypothyroid patients on levothyroxine. We aimed to study the effect of Ramadan fasting on thyroid functions in hypothyroid patients taking levothyroxine.

**Methods:**

We did a comprehensive search of 8 databases for Randomized controlled studies (RCTs) and observational studies investigating the effect of Ramadan fasting on thyroid functions in hypothyroid individuals taking levothyroxine. Relevant data was extracted and analyzed. Mean difference (MD) and standard deviation (SD) were used to evaluate the continuous data. Risk ratios (RR) with a 95% confidence interval were used for outcomes constituting dichotomous data. National Institutes of Health (NIH) tools were used to assess the risk of bias.

**Results:**

Fourteen studies met our inclusion criteria, 3 RCTs, and 11 observational studies, all designed as pre-post studies. Ramadan fasting was associated with a statistically significant increase in TSH in patients who were euthyroid before Ramadan (MD = -0.76 [95% CI; -1.27, -0.25]). However, free thyroxine (FT4) was found to be stable (MD = 0.01, [95% CI; -0.03, 0.06]). All timing points were associated with a significant increase in TSH levels after Ramadan, pre-iftar (MD = -0.69 [95% CI; -1.03, -0.36]), post-iftar (MD = -0.76 [95% CI; -1.12, -0.39]), and pre-suhoor (MD = -1.19 [95% CI; -2.18, -0.19]).

**Conclusion:**

TSH increases significantly after Ramadan. No timing point has superiority in maintaining thyroid control. However, choosing the timing should be individualized according to the patient’s preference to guarantee the most possible compliance.

**Supplementary Information:**

The online version contains supplementary material available at 10.1007/s11845-023-03526-z.

## Introduction

Millions of adult Muslims worldwide observe fasting during the holy month of Ramadan each year. Ramadan fasting is a type of intermittent fasting that is considered an obligatory Islamic tradition, as it is one of the five core pillars of Islam. During Ramadan, observant Muslims abstain from food, liquids, smoking, and sexual intercourse from dawn to dusk for 29–30 days annually. Ramadan, the ninth Islamic month, advances by 10 days each year according to the lunar calendar (355 days a year). Therefore, the number of fasting hours may vary depending on the season. During these fasting hours, Muslims often eat two meals a day: "Iftar", the meal that marks the conclusion of their fast at sunset, and "Suhoor", a quick supper eaten just before dawn [1–3].

The Quran exempts many cases from fasting, including those with chronic medical illnesses that fasting would worsen and those on drug regimens that might be harmed. Nevertheless, many people choose to observe the fast, often against medical advice, and frequently skip their midday doses since taking oral drugs during the fasting period invalidates the fast [4–6]. This has ramifications for a variety of chronic diseases, including the prevalent endocrine illness hypothyroidism [7].

Levothyroxine, the primary medication for hypothyroidism, is primarily absorbed in the small intestine, notably through the ileum [8]. The standard dose of levothyroxine is 1.5 µg / kilogram body weight to be taken 30–60 min before breakfast or 3 h or more after the evening meal on an empty stomach (usually at bedtime) to prevent interference with its intestinal uptake by food or medications [7, 9, 10]. These facts explain the low adherence of hypothyroid patients to the drug [11].

In Ramadan, hypothyroid patients, with the guidance of their medical care providers, have to choose the appropriate timing of levothyroxine intake to ensure full compliance, keeping in mind the precautions that must be taken, such as taking the drug on an empty stomach (no food or beverages for 3–4 h before, and 1 h after) [9]. They may take it before the iftar meal, after iftar, or before suhoor. Adhering to the recommended regimen during Ramadan represents a significant challenge to hypothyroid patients since it is notably difficult to delay the sunset meal (iftar), which is a significant social event and a time of acute hunger, for 30 to 60 min after levothyroxine administration. Additionally, they have relatively few hours in which they are allowed to eat and drink, making it challenging for them to abstain from eating for 3 to 4 h after the iftar meal or before the suhoor meal. Furthermore, most patients are not able to get up 30 min before each predawn suhoor meal for levothyroxine administration [12–14].

This issue has been a field of interest in the last decade, with several studies yielding considerable controversies in their results. Some studies have tested the effect of Ramadan fasting on thyroid functions, while others have compared different time points for levothyroxine intake to choose the optimal timing [12–25]. Although millions of Muslims with hypothyroidism often fast during Ramadan, there is a lack of high-quality evidence on how it affects thyroid functions (TF) and how to manage hypothyroid patients during Ramadan. To our knowledge, this is the first systematic review and meta-analysis pooling the current evidence in this area.

## Methods

This systematic review was performed in accordance with the Preferred Reporting Items for Systematic Review and Meta-Analyses (PRISMA) statement [26].

### Data source and search terms

We searched 8 databases: PubMed, Scopus, Web Of Science (WOS), Ovid, Science Direct, Cochrane Library, Google Scholar, and ClinicalTrial.gov in October 2022. This search was supported by extensive manual searches till the end of November 2022. Without any restrictions or filters, we used these search terms; (Ramadan OR Islamic) AND (Levothyroxine [MeSH Terms] OR Thyroid [MeSH Terms] OR hypothyroidism OR TSH). The detailed search strategy and results can be found in the Supplementary Table 1.

### Eligibility criteria

RCTs and observational studies written in English with the following PICO criteria were included: Population (P): hypothyroid patients taking thyroid hormone replacement; Intervention (I): Ramadan fasting;Control (C): No fasting;Outcome (O): the difference in serum TSH levels before and after Ramadan, and the effect of the drug timing (pre-iftar, post-iftar, and pre-suhoor) on the TSH level change (µIU/mL), the change in free thyroxine (FT4 ng/dl), and the difference in the number of patients with a TSH level above the reference range before and after Ramadan. Studies investigating fasting forms rather than Ramadan or Islamic fasting were not eligible. We excluded animal studies, reviews, pilot studies, post hoc analyses, and studies in a non-English language.

### Study selection

We exported the studies to Rayyan software, removed duplicates, then started title and abstract screening [27]. Each study was assessed by 2 independent reviewers. Six reviewers participated in this step (MMB, ARY, NAE, HB, MAQ, and AAM). Studies that were found relevant after the title and abstract screening were subjected to a more detailed full-text screening according to our prespecified eligibility criteria. Any disputes were resolved by a third reviewer (EMR).

### Data extraction and outcomes

Data from the final included studies were extracted into Microsoft Excel spreadsheets. The extracted data included the following: (a) Summary of the included studies (year, country, sample size, population, study design, and main outcomes measured), (b) Baseline characteristics of enrolled patients. (c) Details of regimens in studies comparing different time points of hormone intake, (d) The primary outcomes were the difference in serum TSH levels before and after Ramadan, and the effect of the drug timing (pre-iftar, post-iftar, and pre-suhoor) on the TSH level change (µIU/mL). The secondary outcomes were the change in FT4 (ng/dl), and the difference in the number of patients with a TSH level above the reference range before and after Ramadan. Each study was subjected to the data extraction step by two independent reviewers and rechecked by a third reviewer for any controversies.

### Risk of bias and quality assessment

Each study was assessed for quality by two independent reviewers. Seven reviewers participated in this step (EMR, MMB, AAM, ARY, HB, NAE, and MAQ) using the National Institutes of Health (NIH) quality assessment tools for controlled intervention studies, pre-post studies, cohort, and cross-sectional studies, chosen according to their study designs [30]. The first tool was used for randomized controlled studies, the second tool for prospective cohort studies, and the last one for retrospective cohort studies.

### Statistical analysis

We used the Review Manager Software (version 5.4) to perform the meta-analysis. Mean difference (MD) and standard deviation (SD) were used to evaluate continuous outcomes, while risk ratios (RR) with a 95% confidence interval were used to measure dichotomous outcomes. In the case of heterogeneity (I2 > 50% or p-value < 0.1), a random effect model was used; otherwise, a fixed-effect model was adopted, and when the heterogeneity persisted, the leave-one-out test was done [28, 29]. In the analysis of TSH change, a subgroup analysis was done based on the baseline thyroid control status of the patients (total sample (euthyroid & non-euthyroid) and euthyroid subgroup). The different TSH baselines and the different study designs were found to be sources of heterogeneity after sensitivity analysis. Studies had wide variability regarding the baseline TSH; that’s why we analyzed the euthyroid subgroup separately to compare the different time points. We used the data of studies which started with only well controlled (Euthyroid) patients [18, 21, 23, 24]. Three timing points of the drug intake were compared according to the change in TSH after Ramadan. “Pre-iftar” means taking the drug at sunset and waiting for 30–60 min before the iftar meal. “Post-iftar” means taking the drug at least 3 h after the iftar meal with no food or beverages during this period. “Pre-suhoor” means taking the drug 30–60 min before the suhoor meal while fasting for at least 3 h before the drug intake. El-Kaissi et al. [17] study was not included in the analysis of TSH change because of the unavailability of data, but it was included in the analysis of the number of patients with TSH levels above the reference ranges. A funnel plot was generated with Review Manager Software (version 5.4) to assess publication bias.

## Results

### Search results

**Fig. 1:**
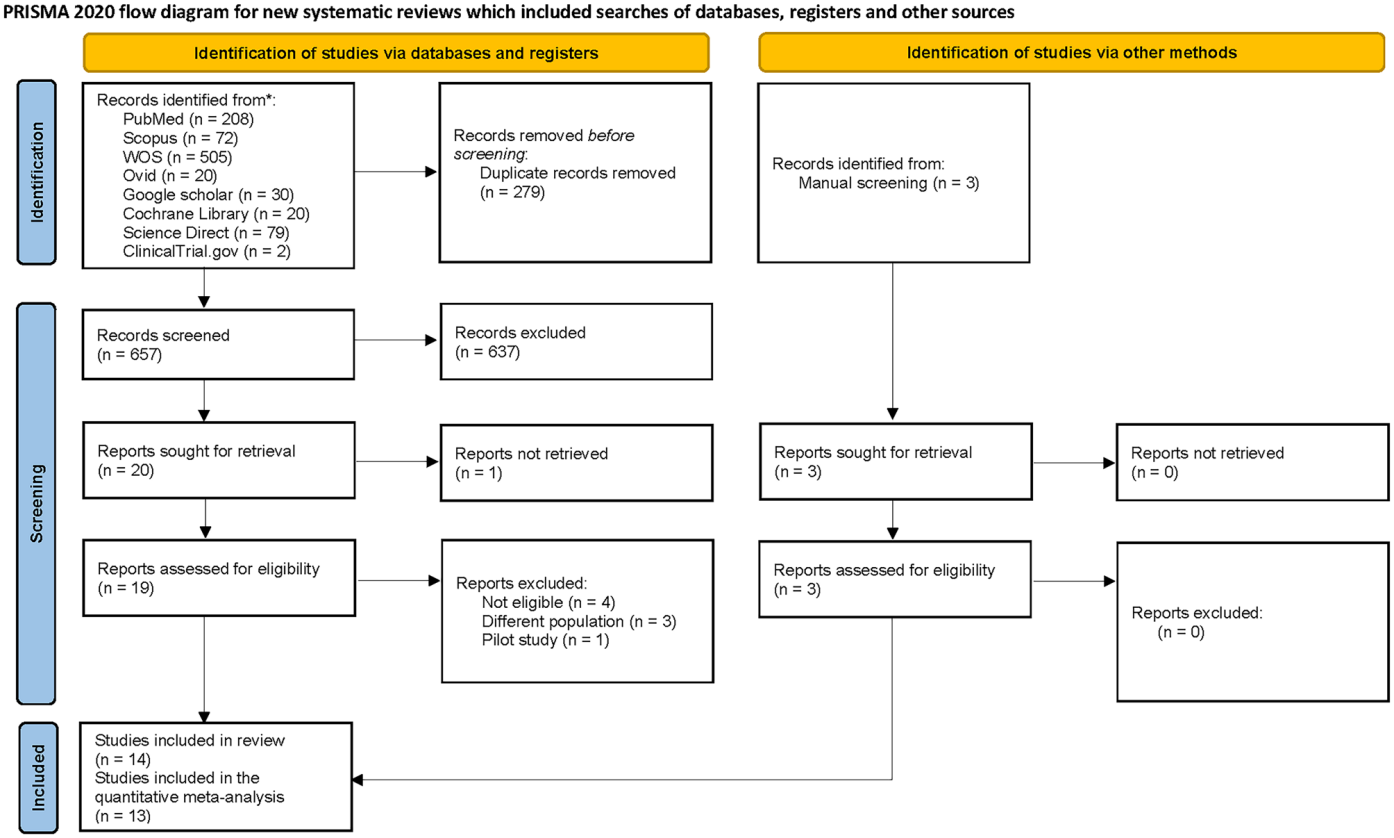
The PRISMA flow diagram

A total of 657 references were screened after removing the duplicates. Twenty studies were found relevant and sought for retrieval and full-text screening. Eight studies were found ineligible to our pre-specified criteria and were excluded. One study was excluded because of the unavailability of its full text [15]. 3 studies were added by manual search. A total of 14 studies were found eligible and included in our systematic review. The PRISMA flow chart is shown in Fig. 1.

### Study characteristics

We included a total of 14 studies with a total of 1661 hypothyroid patients. All studies were comparing the thyroid functions before and after Ramadan (pre-post studies) with differences in the methodology (RCTs, prospective cohort, and retrospective cohort studies). Three studies were randomized controlled trials [17, 21, 23], 11 studies were observational [12–14, 16, 18–20, 22, 24, 25, 31]. Only 13 studies were included in the quantitative analysis as Karoli et al. did not report on any eligible data for this meta-analysis [20.]. All enrolled patients were hypothyroid and on daily levothyroxine doses. Five studies recruited only the well-controlled thyroid patients (euthyroid) [18, 21, 23–25], and the remaining studies didn’t specify a certain group of interest regarding the baseline thyroid status (Euthyroid and Non-euthyroid). Elsherbiny et al. is an extension of Elsherbiny et al. in which data from euthyroid patients only were included and analyzed [18, 19]. The dates of patient recruitment in the studies went from 2012 to 2020. Studies were conducted in different countries: Egypt, United Arab Emirates, Saudi Arabia, Pakistan, Qatar, Turkey, Iraq, Iran, India, and Morocco. The summary of the studies included is shown in Table 1. Most of the patients were females with a mean age of 43 years. Details of baseline characteristics of enrolled patients are provided in Table 2*.* Eight studies compared 2 or more timing points and investigated the effect of timing of levothyroxine intake on thyroid functions after Ramadan [14, 17–19, 21–24]. Definitions of time points in each group were slightly different among the studies which can be attributed to the variability in fasting hours, lifestyles, dietary habits, and prayer time. Details of these studies are shown in Table 3.

**Table 1:** Summary of the included studies

**Study ID**	**Year of recruitment**	**Country**	**Population**	**Sample size (n)**	**Study design**^**b**^	**Main Outcomes measured**
**Al-Qahtani et al.** [21]	2021	Saudi Arabia	Patients with Hypothyroidism^a^	87	Prospective randomized trial	TSH, FT4, weight, and BMI
**El-kaissi et al.** [17]	2019	United Arab Emirates	Patients with Hypothyroidism	148	Prospective randomized trial	TSH and FT4 (pre- and post-Ramadan)
**Ghaffar et al.** [22]	2019	Pakistan	Patients with Hypothyroidism	44	Prospective cohort study	TSH, Triglycerides, Total cholesterol, weight, and blood pressure
**Sheikh et al.** [12]	2016	Pakistan	Patients with Hypothyroidism	64	Prospective cohort study	TSH (pre- and post-Ramadan), patients’ quality of life
**Dabbous et al.** [23]	2017	Qatar	Patients with Hypothyroidism^a^	96	Prospective randomized trial	TSH, preference, body weight, and blood pressure
**Dellal et al.** [24]	2018	Turkey	Patients with Hypothyroidism^a^	62	Prospective cohort study	TSH, FT3, and FT4
**Zaboon et al.** [14]	2018	Iraq	Patients with Hypothyroidism	50	Prospective cohort study	TSH (pre- and post-Ramadan)
**Pakdel et al.** [16]	2016	Iran	Patients with Hypothyroidism	36	Prospective cohort study	TSH, and FT4
**Alkaf et al.** [31]	2012:2017	United Arab Emirates	Patients with Hypothyroidism	481	Retrospective cohort	TSH, FT4 and FT3
**Koca et al.** [13]	2018	Turkey	Patients with Hypothyroidism	97	Retrospective cohort	TSH, and FT4
**Karoli et al.** [20]	2012	India	Patients with Hypothyroidism	47	Prospective cohort study	TSH
**Elsherbiny et al.** [19]	2018 and 2019	Egypt	Patients with Hypothyroidism	393	Prospective cohort study	Preference, Adherence, and TSH
**Elsherbiny et al.** [18]	2018, 2019, and 2020	Egypt	Patients with Hypothyroidism^a^	292	Prospective cohort study	Preference, Adherence, and TSH
**Oudghiri et al.** [25]	2018 and 2019	Morocco	Patients with Hypothyroidism^a^	56	Prospective cohort study	TSH

*TSH* Thyroid stimulating hormone, *FT4* Free thyroxine, *FT3* Free triiodothyronine, *BMI* body mass index

^a^Euthyroid population

^b^All studies are designed as pre and post studies

**Table 2:** Baseline characteristics of enrolled patients

**Study ID**	**Age, mean (SD)**	**BMI or weight, mean (SD)**	**M/F (%)**	**1st measurement**	**2nd measurement**	**Reasons for levothyroxine initiation**	D**ays of fasting - Hours of fasting**
**Al-Qahtani et al.** [21]	45 (11.9)	Weight: 76.8 (15.4), BMI:29.7 (5.6)	16/84%	2 weeks before Ramadan	2 weeks after Ramadan	74.7% surgery for thyroid cancer, 6.9% surgery for benign goiter, 18.4% Hashimoto’s thyroiditis	27.2 (3.2) days
**El-kaissi et al.** [17]	43.5 (12.4)	_	22/78%	3 months before Ramadan	Within 6 weeks after Ramadan	_	_
**Ghaffar et al.** [22]	32.7 (10.6)	Weight: 67.8 (8.9)	_	Within 3 weeks before Ramadan	Within 2 weeks after Ramadan	_	_
**Sheikh et al.** [12]	44.2 (13.2)	BMI: 30.2 (5.9)	12.5/87.5%	Within 6 weeks before Ramadan	Within 1 to 2 weeks after Ramadan	73.4% Etiology not known, 9.4% Autoimmune, 7.8% Postsurgical, 7.8 Post–radioactive iodine, 1% Congenital	26.5 (3) days 15 h
**Dabbous et al.** [23]	45.1 (12.5)	Weight:80.7 (16), BMI:31.5 (5.9)	11.5/88.5%	2 weeks before Ramadan	2 weeks after Ramadan	Primary hypothyroidism	25.7(3.6) days
**Dellal et al.** [24]	49 (13)	BMI: 29.2 (5.8)	14.5/85.5%	1 week before Ramadan	At the 26-28th day of Ramadan	72.6% Primary / post-radioactive iodine, 27.4% Postoperative	27(3) days 16–17 h
**Zaboon et al.** [14]	44.12 (13.72)	BMI: 32.2 (6.7)	16/84%	1 month before Ramadan	Within one month after Ramadan	Primary hypothyroidism	_
**Pakdel et al.** [16]	Range (12–56)	_	0/100%	3 days before Ramadan	Day 27 of Ramadan, and Two months after Ramadan	Hypothyroidism	> 16 h
**Alkaf et al.** [31]	44 (12.7)	BMI: 30 (6)	10.2/89.8%	3 months Before Ramadan	1–2 weeks, and 3–6 months after Ramadan	Chronic thyroiditis 88.4%, post-surgical 5.2%, Total thyroidectomy and radioiodine ablation for thyroid carcinoma 3.3%, Radioiodine ablation 1.6%, Postpartum thyroiditis 1.2%	14 h
**Koca et al.** [13]	45 (6.3)	_	7.2/92.8%	1 month before Ramadan	Within the month after Ramadan	81 patients had Hashimoto hypothyroidism, 14 had iatrogenic hypothyroidism, and 2 had post-radioactive iodine ablation hypothyroidism.	_
**Karoli et al.** [20]	40.2(13.8)	_	74.4/27.6%	1 week before Ramadan	At the end of Ramadan	Hypothyroidism	_
**Elsherbiny et al.** [19]	Mean 40.2	_	3.6/96.4%	Before Ramadan	Within 6 weeks after Ramadan	74% Hashimoto thyroiditis, 16.8% Thyroid surgery, 2% Radioactive iodine ablation, 5.3% Subacute thyroiditis, 1.9% Postpartum thyroiditis	15 - 15.5 h
**Elsherbiny et al.** [18]	43.5 (13.3)	_	4.1/95.9%	Before Ramadan	Within 6 weeks after Ramadan	73.3% Hashimoto thyroiditis, 15.8% thyroidectomy, 2.4% Radioiodine ablation, 1.4% postpartum thyroiditis, 7.2% unclassified	_
**Oudghiri et al.** [25]	51.4 (18)	_	10.7/89.3%	1 week before Ramadan	1 week after Ramadan	47% Iatrogenic hypothyroidism, 53% Hashimoto Thyroiditis	_

*BMI* body mass index, *M/F* male/female, *LT4* levothyroxine

**Table 3:** Details of the studies investigating different time points of Levothyroxine intake

**Study ID**	**Definition of each group**	**number of patients in each group**	**Conclusion**
**Al-Qahtani et al.** [21]	G1: 30 min pre-iftar G2: 3 h post-iftar G3: 1 h pre-suhoor	G1: 31 G2: 34 G3: 22	Fasting patients who took LT4 pre-iftar did not experience significant changes in TSH, whereas those who took LT4 post-iftar or pre-suhoor did. TSH changes during Ramadan may be associated with age (inverse association), weight gain, and the number of nonadherence to LT4 days.
**El-kaissi et al.** [17]	G1: 30 min pre-Iftar G2: 3 or more hours post-iftar G3: 30 min pre- suhoor meal	G1: 50 G2: 46 G3: 52	Instructing patients to take levothyroxine at the time of breaking the fast 30 min before the Iftar meal minimizes unfavorable changes in plasma TSH post-Ramadan. In contrast, instructing patients to take levothyroxine 3 h post-Iftar or 30 min before Suhur led to a greater rise in post-Ramadan TSH.
**Dabbou et al.** [23]	G1: 30 min pre-iftar G2: 30 min pre-suhoor	G1: 50 G2: 46	Choosing an optimal time for levothyroxine intake during the month of Ramadan remains a challenge. The current study did not provide any evidence on ideal time and dose of levothyroxine administration during fasting to manage hypothyroidism.
**Dellal et al.** [24]	G1: Late evening at 22.30–23.00 pm before sleep (post-Iftar 2 to 3 h) G2: 01:30–03:00 am at least 30 min pre-suhoor	G1: 18 G2: 44	The increase in TSH was not significant after Ramadan. While there was an insignificant increase in median TSH, about one-third of patients had lower TSH, indicating the need to evaluate every patient individually and follow closely during Ramadan.
**Zaboon et al.** [14]	G1: at least 30 min pre-iftar G2: 2 h post-iftar G3: 1 h pre-suhoor	G1: 20 G2: 10 G3: 20	No significant differences in TSH control were observed in patients taking L-thyroxine at pre-iftar, post-iftar, or pre-suhoor time in Ramadan.
**Elsherbiny et al.** [19]	G1: At sunset pre-iftar (1 h) G2: 3–4 h post-iftar G3: Before starting to fast at sunrise	G1:159 G2: 144 G3: 19	The first and second regimens or a combination of both was preferred by most patients. High rates of adherence and post-Ramadan euthyroidism were observed. Adherence to the preferred regimen is the main determinant of post-Ramadan euthyroidism.
**Elsherbiny et al.** [18]	G1: At sunset pre-iftar (1 h) G2: 3–4 h post-iftar G3: Before starting to fast at sunrise	G1: 101 G2: 127 G3: 14	Fasting Ramadan in well controlled hypothyroid patients resulted in a significant increase in post-Ramadan TSH, yet 80% of the patients remain euthyroid after Ramadan. Post-Ramadan TSH and euthyroidism are related to adherence and pre-Ramadan TSH.
**Ghaffar et al.** [22]	G1: ≥ 30 min pre-suhoor G2: < 30 min pre-suhoor G3: Post or with suhoor	G1: 18 G2: 12 G3: 14	There is an increase in TSH in participants taking thyroxine less than 30 min before suhoor with suhoor or post suhoor and there is a positive correlation between TSH levels and lipid parameters.

All regimens are conditioned on refraining from food at least 3 h before the intake and at least 30 min after

*G* group or regimen, *ft4* free thyroxine, *TSH* thyroid stimulating hormone, *LT4* levothyroxine

### Quality assessment

By using NIH quality assessment tools [30], all studies were found to have fair quality. The details of each domain are provided in Supplementary Tables 2, 3, and 4.

### Quantitative results

#### Change in TSH

**Fig. 2:**
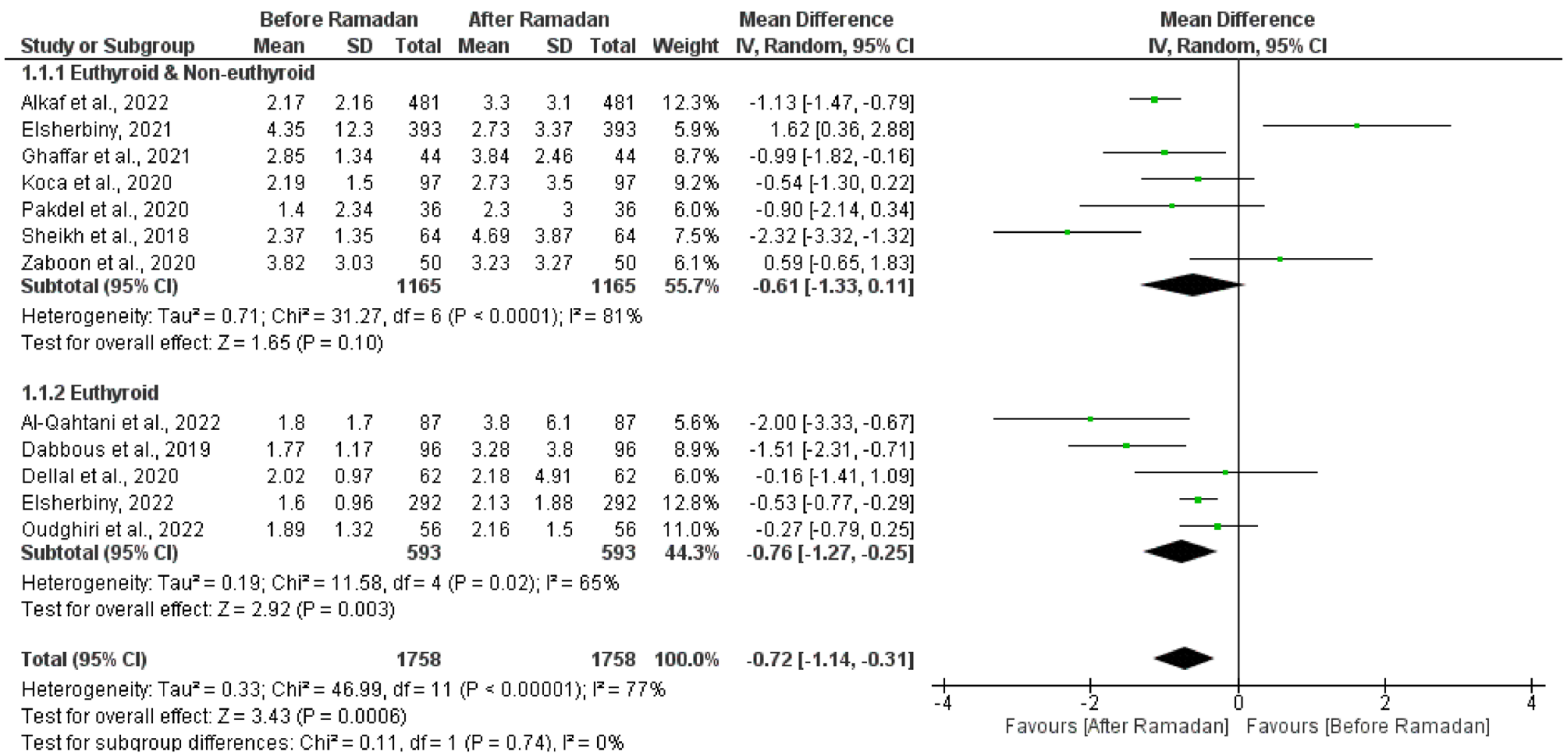
Forest plot showing the TSH before and after Ramadan with subgrouping according to the baseline thyroid status (Euthyroid) and (Euthyroid & Non - Euthyroid)

**Fig. 3:**
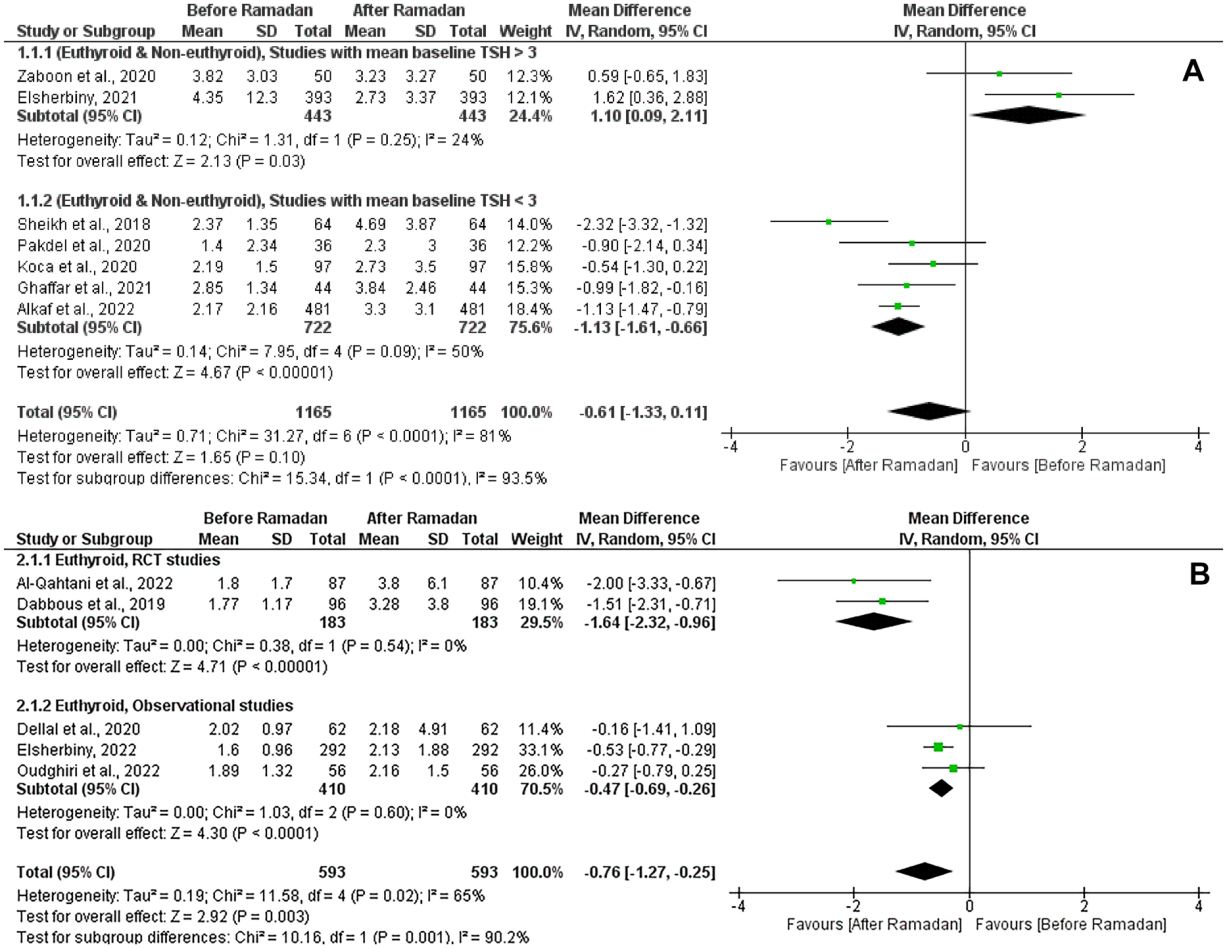
**A** Forest plot showing subgroup analysis of (Euthyroid & Non - Euthyroid) studies according to the TSH level (< 3 - > 3). **B** Forest plot showing subgroup analysis of (Euthyroid) studies according to the study designs (RCTs - Observational)

The test for the overall effect of 12 studies showed a significant increase in TSH after Ramadan (MD = -0.72 [95% CI; -1.14, -0.31], p = 0.0006). The significance was attributed to the euthyroid subgroup (MD = -0.76 [95% CI; -1.27, -0.25], p = 0.003). Substantial heterogeneity was observed in both groups (I^2^ = 81%, and 65% respectively) (Fig. 2). No publication bias was observed, as shown in Supplementary Fig. 1. Another subgroup analysis was performed based on the mean baseline TSH level in the (euthyroid and non-euthyroid) group, studies with mean TSH > 3 at the baseline showed a significant decrease in the TSH after Ramadan (MD = 1.1 [95% CI; 0.09, 2.11], p = 0.03). However, studies with mean TSH < 3 at the baseline showed a significant increase in the TSH after Ramadan (MD = -1.13 [95% CI; -1.61, -0.66], p < 0.00001). No significant heterogeneity was observed in both groups (I^2^ = 24%, and 50% respectively) (Fig. 3A).

When examining the effect of study design on studies that included euthyroid patients only at the baseline, RCTs and observational studies showed a significant increase in the TSH after Ramadan in both groups (MD = -1.64 [95% CI; -2.32, -0.96], p < 0.00001), and (MD = -0.47 [95% CI; -0.69, -0.26], p < 0.0001) respectively. We observed no significant heterogeneity between the studies in both groups (I^2^ = 0%, and 0%) (Fig. 3B).

#### Comparison of 3 timing points of levothyroxine intake in euthyroid patients

**Fig. 4:**
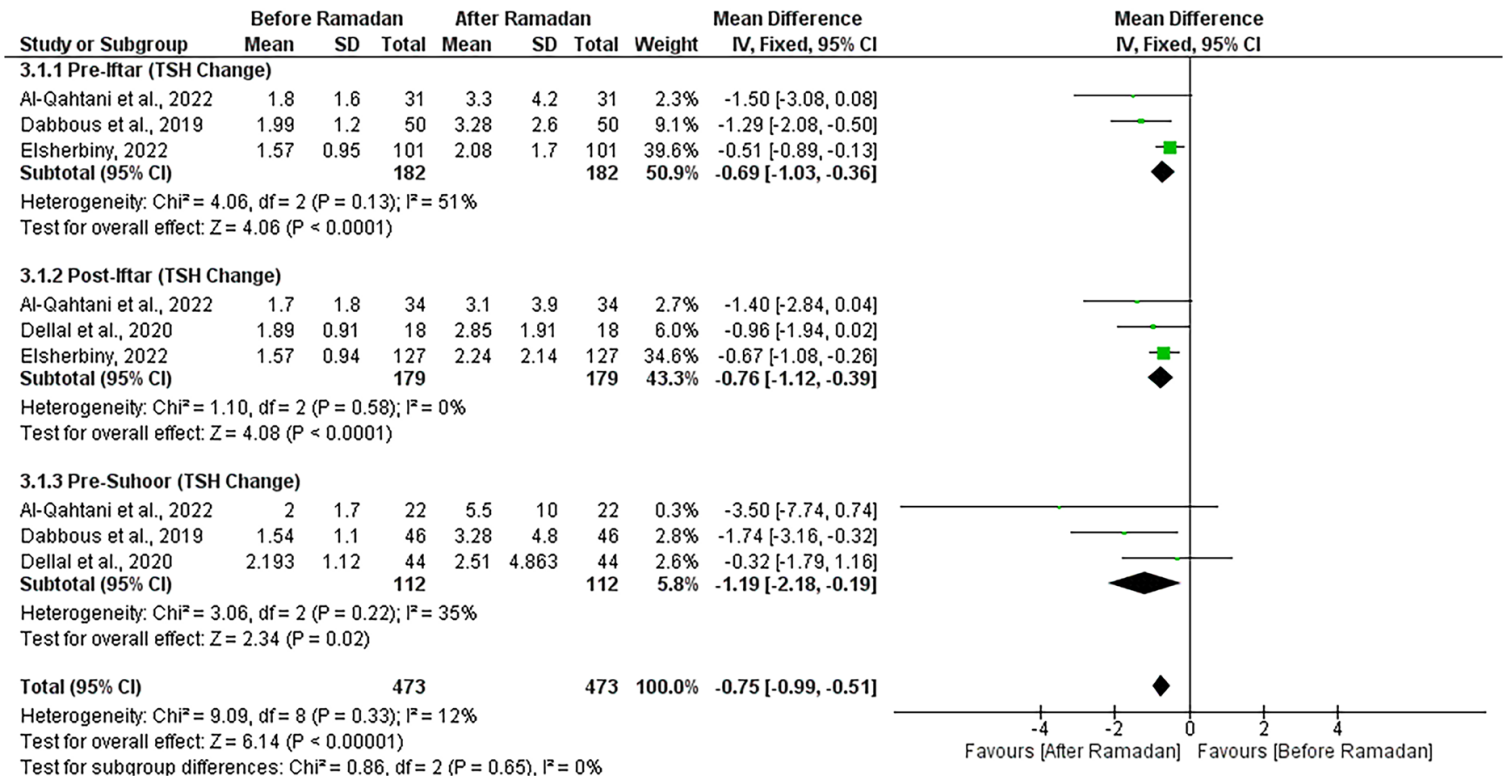
Forest plot showing the effect of timing of levothyroxine intake on TSH change

The pooled effect of 4 studies showed that all time points of levothyroxine intake were associated with a significant increase in TSH levels after Ramadan in euthyroid patients [18, 21, 23, 24]. Pre-iftar (MD = -0.69 [95% CI; -1.03, -0.36], p < 0.0001), post-iftar (MD = -0.76 [95% CI; -1.12, -0.39], p < 0.0001), pre-suhoor (MD = -1.19 [95% CI; -2.18, -0.19], p = 0.02). No significant heterogeneity was observed (I^2^ = 12%, P = 0.33) (Fig. 4).

#### Number of patients with TSH above the reference range

**Fig. 5:**
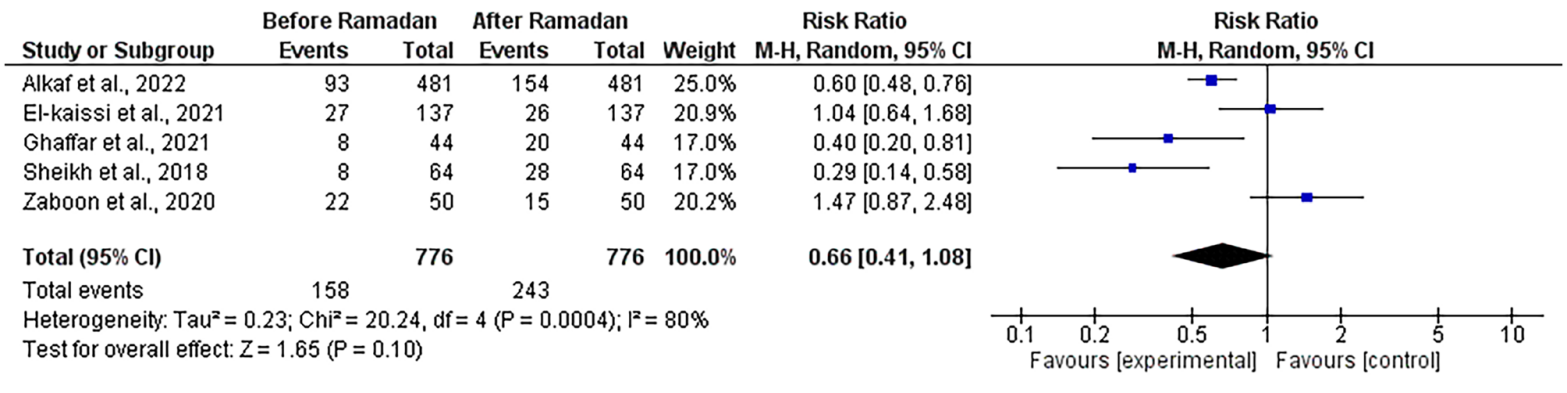
Forest plot showing the number of patients with TSH above the reference range before and after Ramadan

The pooled effect of five studies [12, 14, 17, 22, 31] showed that the number of patients with TSH levels above the reference ranges was comparable with an insignificant increase after Ramadan (RR = 0.66, [95% CI; 0.41, 1.08], P = 0.1) with significant heterogeneity not solved by the random effects model or leave-one-out test (I^2^ = 80%, P = 0.0004) (Fig. 5).

#### Free Thyroxine (FT4) change

**Fig. 6:**
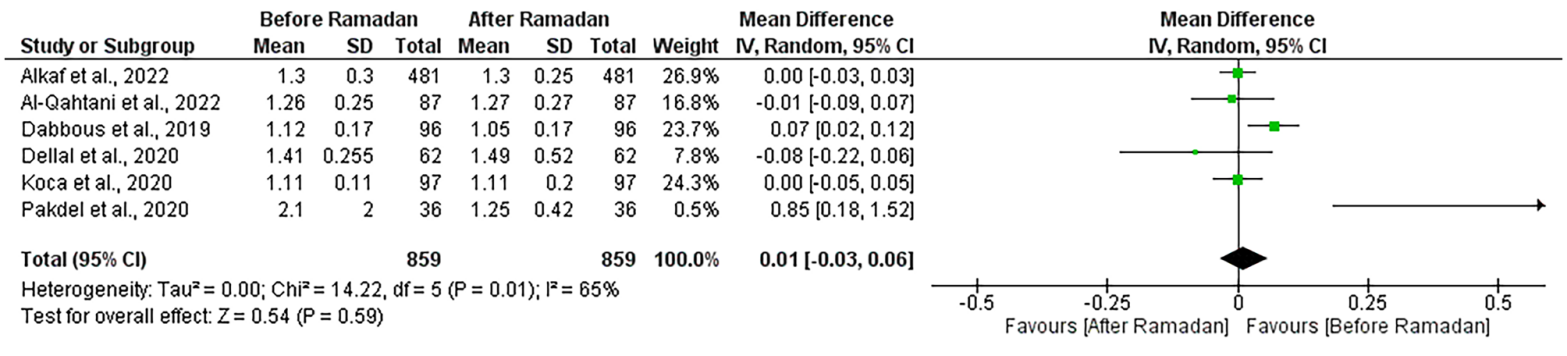
Forest plot showing the free thyroxine (FT4) change before and after Ramadan

This analysis comprised six studies [13, 16, 21, 23, 24, 31]. Free thyroxine (FT4) was found to be stable after Ramadan with no significant difference (MD = 0.01, [95% CI; -0.03, 0.06], p = 0.59). Heterogeneity was addressed (I^2^ = 65%, P = 0.01), and also not resolved by the random effects model or leave-one-out test (Fig. 6)*.*

## Discussion

This study aimed to investigate the effect of Ramadan fasting on the thyroid status of patients taking levothyroxine, which was an area of interest for research, considering that Ramadan fasting is a month-long period of fasting experienced by millions of Muslims around the world each year. Our meta-analysis showed that Ramadan fasting significantly increases TSH levels in patients taking levothyroxine. However, subgroup analysis demonstrated this significant change specifically in patients with well-controlled baseline thyroid status (euthyroid) only. On the other hand, the meta-analysis of the studies that did not specify the thyroid status at baseline found an insignificant increase in TSH after Ramadan. Differences in baseline TSH and study designs were found to be the most important sources of the observed heterogeneity. Based on the sensitivity analysis, we further sub-grouped the studies accordingly. Studies with mean TSH > 3 at the baseline showed a significant decrease in the TSH after Ramadan, which can be explained by the fact that those patients might have gotten more care during Ramadan to control their thyroid status towards lower TSH levels. The meta-analysis of the studies that considered different time points of drug intake showed that timing, per se, did not affect the change in TSH levels. TSH increased in euthyroid patients after Ramadan to nearly the same degree, regardless of the time of drug intake. However, our analysis did not show a significant increase in the number of patients with TSH above the reference ranges. In addition, no significant effect of fasting on T4 levels was found.

Fasting during Ramadan causes some transient metabolic disturbances that can explain the changes in pharmacodynamics and pharmacokinetics of different drugs. Glucose increases mainly through increased gluconeogenesis in early fasting whereas triglycerides and cholesterol levels are affected by increased ketogenesis later [23–33]. Metabolism, especially carbohydrate and lipid metabolism, has a mutual influence relationship with TSH [34–36]. The nature of food consumed during Ramadan differs from other times according to Muslim customs and traditions [37]. The nature of diet can affect TSH levels [38]. Fasting, in addition, can affect body weight, which in turn has a mutual relationship with TSH levels [39]. Moreover, the physical and mental stress associated with fasting can affect TSH levels [40]. All these hypotheses can explain the changes in drug effectiveness during and after Ramadan, together with the fact that Ramadan fasting can affect patients’ compliance due to the limited hours in which they can eat and drink and the changes in their drug schedules, especially in patients taking multiple drugs. So, it has been a focus of research to study the effect of Ramadan fasting on thyroid functions, especially in hypothyroid patients, and to identify the measures by which they can maintain good thyroid control.

The most recent study, by Elsherbiny et al., found that Ramadan fasting was associated with a significant increase in TSH, which is consistent with the results of most studies included in this meta-analysis [18]. On the other hand, Zaboon et al., Dellal et al., and Pakdel et al. found that TSH levels were comparable before and after Ramadan [14, 16, 24]. Only Pakdel et al. showed a significant decrease in T4 levels. Oudgheri et al. and Elsherbiny et al., reported that 19–20% of the euthyroid patients before Ramadan turned into a dysthyroid state after Ramadan [18, 25]. This percentage was higher in other studies, including 32% in El-Kaissi et al., and 40% in Zaboon et al. [14, 15].

Choosing the optimal timing of drug intake, whether in “pre-iftar”, “post-iftar”, or “pre-suhoor” was shown to be a major controversy among the included studies. Al-Qahtani et al. concluded that taking the drug at sunset 30 min before iftar was found the least timing to affect TSH after Ramadan, which aligns with the conclusion of El-Kaissi et al., as they found no significant changes in TSH on “pre-iftar” regimen [17, 21]. In Elsherbiny et al., euthyroidism was more evident in patients who chose to take the drug 60 min before, or 3–4 h after the iftar meal. While Dabbous et al., and Sheikh et al. found that TSH change was not affected by the timing of LT4 intake, which is consistent with our finding in this meta-analysis [12, 23]. On the other hand, Dellal et al. and Zaboon et al. found no significant TSH change in all groups [14, 24]. These inconsistencies can be explained by different rates of adherence and compliance of the patients to the regimens and the precautions. Karoli et al. found that non-adherence in keeping at least a 2-h interval between meals and levothyroxine intake was significantly correlated to the variations in TSH [20]. Oudghiri et al. and Elsherbiny et al. reported the highest adherence rates and highlighted the significant association between adherence and maintaining euthyroid state [18, 19, 25]. This raises the concern of adherence as the most important factor to guarantee good thyroid control during and after Ramadan. This can be achieved by interplay between the caregiver and the patients, aiming to tailor the regimens according to their lifestyles and time schedules during Ramadan days. Other strategies have been postulated to increase compliance in patients, like changing the regimen from daily to weekly, especially in patients where compliance is a major issue [41]. Twice or thrice-per-week regimens were also compared with the standard daily dosing during Ramadan in a pilot study, and it showed promising results that should be investigated further in the future [42].

To our knowledge, this is the first systematic review and meta-analysis pooling the evidence on this point. There are a few limitations to our study; including the observational nature of most of the included studies, and the combination of RCTs and observational studies can represent a potential bias. The included studies were found to be of fair quality based on the risk of bias tools used, that’s why we cannot build a strong evidence in this area based on these results alone. Also, heterogeneity was noticed among the studies in different aspects like different TSH baselines, different indications of thyroid hormone replacement, different definitions of timing of the drug intake, different recommendations given to the patients, the different number of days fasted by the patients, different fasting hours, different TSH measuring time, and lastly and most importantly, different adherence rates. All these variabilities were supposed to be the source of the statistical heterogeneity observed in the results. We tried to address these issues by subgrouping and choosing the data that corresponds to the same baselines as much as possible. The hours of fasting can range from 10 to 20 h according to the country and the year. However, in our included studies, the maximal time difference is 4.5 h between the most eastern longitude (India) and the most western longitude (Morocco). So, the fasting hours did not differ that much, ranging from 14 to 16 h [20, 25].

In conclusion, Ramadan fasting is associated with a significant increase in TSH levels. However, achieving TSH levels above the reference range after Ramadan is not significant. Free thyroxine levels were also found to be stable after Ramadan. Timing per se (pre-iftar, post-iftar, or pre-suhoor) doesn't affect the change in TSH. No sufficient evidence supports increasing the dose of levothyroxine during or after Ramadan. However, management should be individualized. The compliance of the patients who intend to fast to the regimens and the precautions should be greatly emphasized by the medical care providers. Due to the small number of studies included, the fair quality, and the observational nature of most of the studies, more research efforts are still warranted to justify the management plan for the hypothyroid patients during Ramadan.

### Supplementary Information

Below is the link to the electronic supplementary material.

Supplementary file1 (DOCX 28 KB)
